# Brain activity studied with magnetic resonance imaging in awake rabbits

**DOI:** 10.3389/fnimg.2022.965529

**Published:** 2022-09-07

**Authors:** Craig Weiss, Nicola Bertolino, Daniele Procissi, John F. Disterhoft

**Affiliations:** Feinberg School of Medicine, Northwestern University, Chicago, IL, United States

**Keywords:** BOLD, Alzheimer model, resting state connectivity, intrinsic functional connectivity, circadian rhythm, cholesterol, MEMRI, ICA

## Abstract

We reviewed fMRI experiments from our previous work in conscious rabbits, an experimental preparation that is advantageous for measuring brain activation that is free of anesthetic modulation and which can address questions in a variety of areas in sensory, cognitive, and pharmacological neuroscience research. Rabbits do not struggle or move for several hours while sitting with their heads restrained inside the horizontal bore of a magnet. This greatly reduces movement artifacts in magnetic resonance (MR) images in comparison to other experimental animals such as rodents, cats, and monkeys. We have been able to acquire high-resolution anatomic as well as functional images that are free of movement artifacts during several hours of restraint. Results from conscious rabbit fMRI studies with whisker stimulation are provided to illustrate the feasibility of this conscious animal model for functional MRI and the reproducibility of data gained with it.

## Introduction

Magnetic resonance imaging (MRI) has been used to examine brain-wide structural and functional properties of the brain non-invasively in humans and experimental animal subjects. Contrast in MRI is based on differences in water content of different tissues and subregions, and differences in the paramagnetic properties of oxygenated and deoxygenated hemoglobin are used to examine functional properties of the brain. This blood oxygen level dependent (BOLD) contrast of tissue forms the basis of functional MRI. An important requirement for accurate localization of functional changes is that subjects remain immobile during image acquisition. Stability has been achieved by instructing humans not to move, by anesthetizing experimental animal subjects to induce immobility, or by prolonged and repeated habituation sessions for awake animal subjects. Although mice and rats are common experimental subjects for neuroscience research, they do not tolerate restraint well without extensive habituation and with frequent rewards for immobility [although read the articles by Ferris (2022) and by Russo et al. (2021) in this Research Topic for use of the rat in imaging research]. Non-human primates have been used after extensive habituation and training on goal directed tasks, but they are scarce and expensive to use. An alternative is to use the rabbit, a prey species that has evolved to become immobile in response to a potential threat in a confined space.

**Figure 1:**
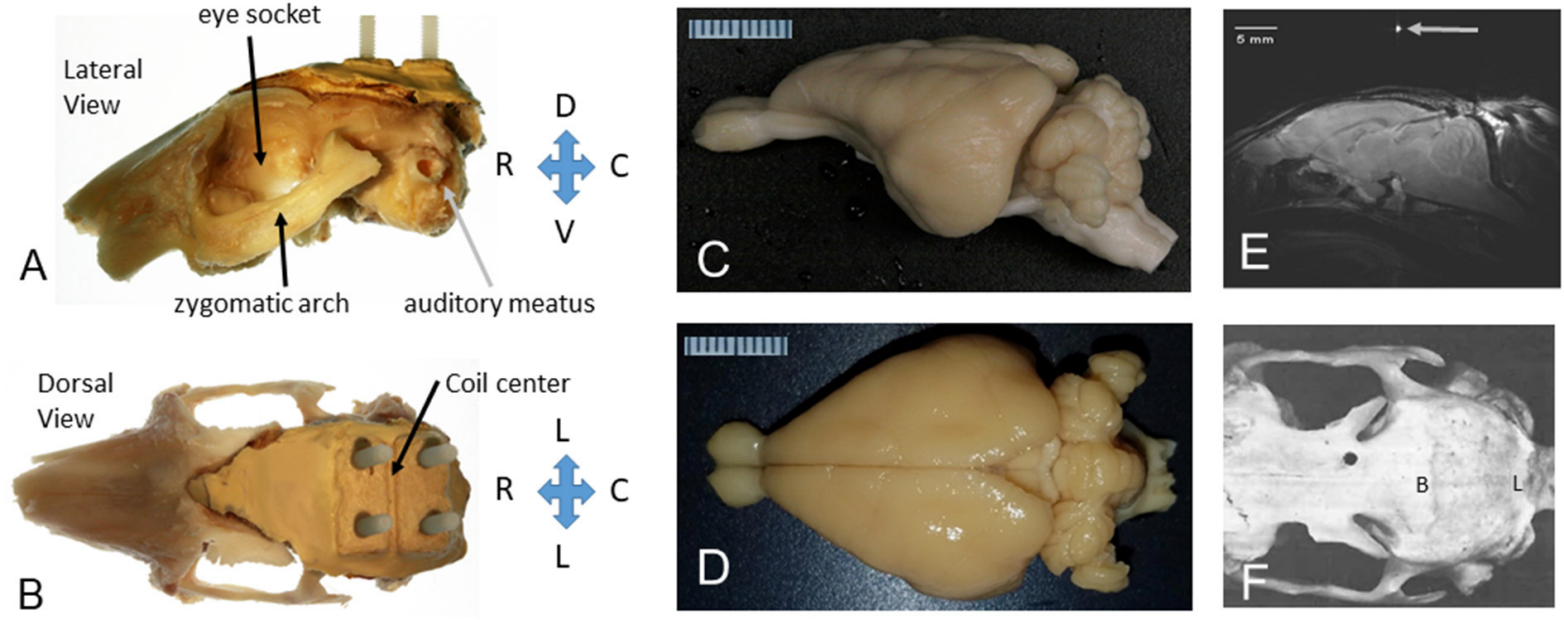
The rabbit skull and brain as prepared for MRI in stereotaxic space. **(A)** Lateral view of the left side of the rabbit skull with a headbolt attached to the top of the skull. The orbit (eye socket), zygomatic arch, and auditory meatus are shown for reference. The “headbolt” was premade by encasing four nylon machine bolts (6–32 × ¾″) with a batch of dental acrylic (Weiss et al., 2018). **(B)** Dorsal view of the rabbit skull with a headbolt attached to the top of the head. The mold for the headbolt included a ridge that formed a valley to hold a capillary tube filled with water to mark the center of the headbolt, as shown in **(E)**. **(C)** Excised rabbit brain from a lateral perspective. **(D)** Excised rabbit brain from a dorsal perspective. A millimeter scale is shown in **(C,D)**. **(E)** A midsagittal slice through the rabbit brain showing the position of the brain after the image was leveled to the stereotaxic standard for the rabbit brain, i.e., with lambda 1.5 mm below bregma. **(F)** Dorsal view of a denuded rabbit skull (rostral to the left). Bregma **(B)** is indicated as the intersection of the sagittal bony suture and the coronal bony suture. Adapted from Weiss et al. (2018).

This review is written to highlight that the awake rabbit tolerates restraint very well which obviates the need for sedation or anesthesia during awake imaging sessions, it has a strong skull that can support a head fixation device, it is not nocturnal which avoids confounds with circadian rhythms (see further discussion below), it has a relatively smooth cortex (Figure 1) which facilitates localization of activated voxels, a differentiated caudate and putamen which is similar to that of humans (and unlike the rodent brain), and for those studying Alzheimer's Disease, the rabbit has the same amino acid sequence for amyloid as found in humans (Davidson et al., 1992), a lipid physiology (which interacts with amyloid) that is also similar to that of humans (Fan et al., 2015), and similar interactions of cholesterol and amyloid (Ghribi et al., 2006; Schreurs and Sparks, 2016).

Rabbits are readily available from approved vendors, and although they are a “covered” species, i.e., their care is governed by the Animal Welfare Act of 1966 (and they are not excluded by the 2002 amendment to the law that affected mice, rats, and birds), the required documentation for covered species is in keeping with good laboratory practices. There are also established handling techniques for working with rabbits, and specialized acrylic or soft wrap restrainers for the handling and restraint of rabbits are commercially available.

An awake, immobile animal can be used to acquire artifact free MRI signals and can also be used to determine intrinsic functional connectivity among different brain regions, i.e., resting state connectivity by detecting correlations across time in the BOLD signals.

The experimental setup requires a 20 cm inner diameter gradient coil, which is commercially available for the same cost as the 12 cm gradients commonly used to image rodents. It is possible to use a rat surface coil as a receiver and a volume coil as transmitter. Although the 20 cm gradient coil has a lower strength and slew rate as compared to the smaller gradient, this shortcoming is compensated by the larger brain size of the rabbit requiring a lower resolution leading to a boost in signal to noise ratio. The BOLD signal has been used widely to examine correlated neural activity across regions in human and rodent brains (Xu et al., 2022), and as a result of aging (Hrybouski et al., 2021; Tsvetanov et al., 2021). The fMRI time course can also be analyzed in regard to an event by analyzing related changes in BOLD activity that are time locked to a stimulus or to a movement. We have used these event-related analyses to examine responses in the visual cortex and the lateral geniculate nucleus to flashes of green light (Wyrwicz et al., 2000), and in the somatosensory cortex and ventral posterior thalamus to examine responses to whisker vibration (Schroeder et al., 2016).

Here we review work using the rabbit to examine intrinsic functional connectivity, event related activations, and brain behavior relationships due to learning, especially during classical conditioning of the eyeblink reflex, as assayed with fMRI and with activity induced changes in T1-weighted signals following administration of manganese chloride to label activated neurons. Much of the reviewed work is from our previously published work using a 7T/30 cm horizontal bore magnet. We encourage others to consider using the awake rabbit to acquire functional activation of different circuits simultaneously from the entire brain. According to August Krogh's principle of animal selection (Krogh, 1929; Krebs, 1975), one or very few animal species may be ideal for the question being studied. In this regard, the rabbit may be the ideal experimental animal for MRI based experiments, and its nature to become immobile when exposed to a novel situation is the reason we initiated these experiments (Wyrwicz et al., 2000). We found that a single habituation session lasting ~1 h (while running an EPI pulse sequence) is sufficient for rabbits to habituate to the MRI environment and remain immobile enough to complete imaging experiments (Schroeder et al., 2016). We have found that experiments requiring minimal movement on the part of the animal, such as results of sensory stimulation, or eyeblink conditioning can be done without compromising the imaging results due to motion-related artifacts. The major limitation of using the rabbit is the per diem costs which are larger than those of rats or mice. However, in our experience, the purchase price of rabbits, rats, and mice on a per animal basis is quite comparable.

## Anesthesia and restraint

Successful MRI studies require subjects to remain stationary during the imaging session so that the MR signal does not blur between neighboring, or distant voxels. Humans can remain stationary enough for good image acquisition, but most non-human animals require anesthesia or extensive habituation to restraint in order to achieve adequate stability when imaging with 0.5 mm in-plane resolution (our standard for fMRI of rabbit brain). The rabbit is exceptional in this regard, i.e., the species has evolved to become immobile when exposed to new stimuli in a confined space.

Our initial imaging experiments with rabbits relied on use of an atraumatic “headbolt” that was surgically implanted onto the skull during a sterile surgery, and which was used after recovery to immobilize the head in the stereotaxic plane during imaging, or during eyeblink conditioning (Figure 1). The headbolt system was also used to reproduce the positioning of the animal head inside the MRI scanner bore improving the spatial registration of the images between sessions. Accurate registration of the image volumes between sessions is important to obtain high quality results during ICA group analysis.

The headbolt consisted of two or four nylon bolts embedded in dental cement and was sterilized prior to surgery. A detailed description of the headbolt and surgical technique for fixing it to the skull can be found in Weiss et al. (2018). In that article we described the use of Metabond, a cement that holds securely enough to the skull to obviate the need for any skull screws and avoids any risk of damage to the underlying brain. We do, however, place a threaded hole and nylon machine bolt into the sinus over each eye orbit for assurance. The four bolt headbolt was used with an annular send/receive coil; the two bolt headbolt was used with a newer customized three-channel phased array coil (RAPID MR International, Columbus, OH). We used the newer coil to collect the data we presented on memory- and learning-related network connectivity changes in the awake rabbit (Bertolino et al., 2021). We have also used a three-bolt version of the headbolt where one of the bolts was replaced with a plastic guide tube for a removable cannula that was targeted to the lateral ventricle for injection of magnetic nanospheres attached to an antibody (Rozema et al., 2020).

**Figure 2:**
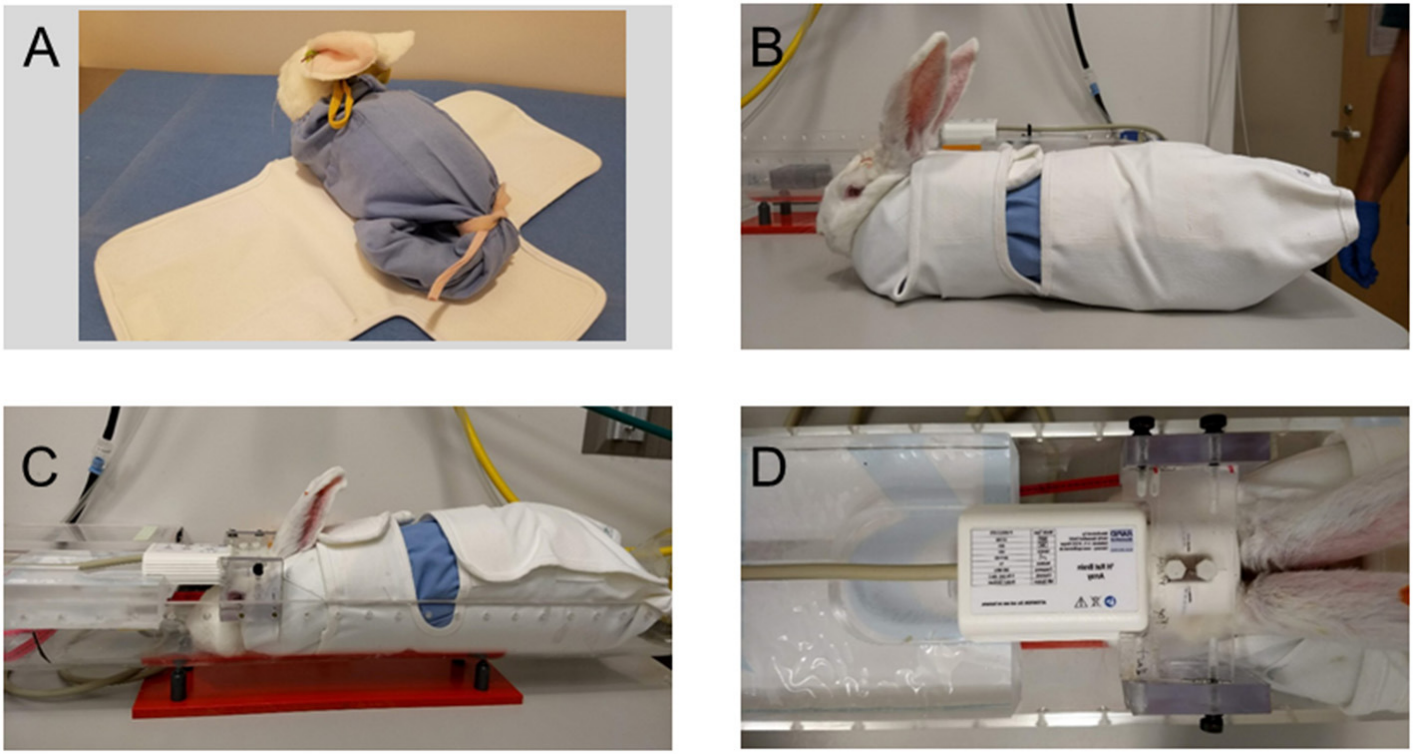
Restraining the awake rabbit for fMRI sessions. **(A)** The rabbit is placed head first into a soft cotton sac with a drawstring at the neck and tail of the rabbit. The cotton sack shown here was made from the leg of a pair of surgical scrub pants. The “bagged” rabbit is then placed upon an outer canvas wrap that secures upon itself with large patches of hook and loop fasteners. **(B)** The canvas wrap is secured snugly around the rabbit to swaddle it in place. **(C)** The rabbit in the canvas wrap is placed within an acrylic cradle. The preamp and an acrylic crossbar are placed upon two nylon bolts that are part of the premade headbolt, and the crossbar is fastened to the sides of the cradle with nylon thumbscrews. **(D)** Another piece of acrylic was machined to accept the back edge of the three-channel phased array coil and its cable. Note that the two nylon anchor bolts are clearly visible within the opening of the coil module. Adapted from Bertolino et al. (2021).

Imaging brain activity without the use of anesthetics is important to visualize normal brain activity (Gao et al., 2017; Ma et al., 2022). A direct comparison of brain activity in the rabbit before and during anesthesia was made by Aksenov et al. (2015). They found that isoflurane, fentanyl, or isoflurane plus fentanyl reduced the area and magnitude that the BOLD signal evoked by whisker stimulation during the awake state, and the response to whisker stimulation differed depending upon the type of anesthesia. An alternative to using anesthetics is to immobilize the head and habituate the animal to restraint. Chang et al. (2016) used 8–10 sessions of habituation for their rat-based studies and acquired good images, and Russo et al. (2021) used 18 sessions across 3 weeks to accustom their rats to the MRI environment. As mentioned already, our rabbits required only a single session of habituation in order to achieve immobility and good image quality. As shown in Figure 2, the rabbit was placed in a soft cotton sac that was placed within a heavier canvas wrap with large sections of hook and loop fasteners to swaddle the rabbit. An acrylic cross bar was used to sandwich the coil in place, and nylon nuts were used to hold the preamplifier in place. Another set of nylon bolts was then used to secure the cross bar to the cradle and the cradle was placed within the magnet.

**Figure 3:**
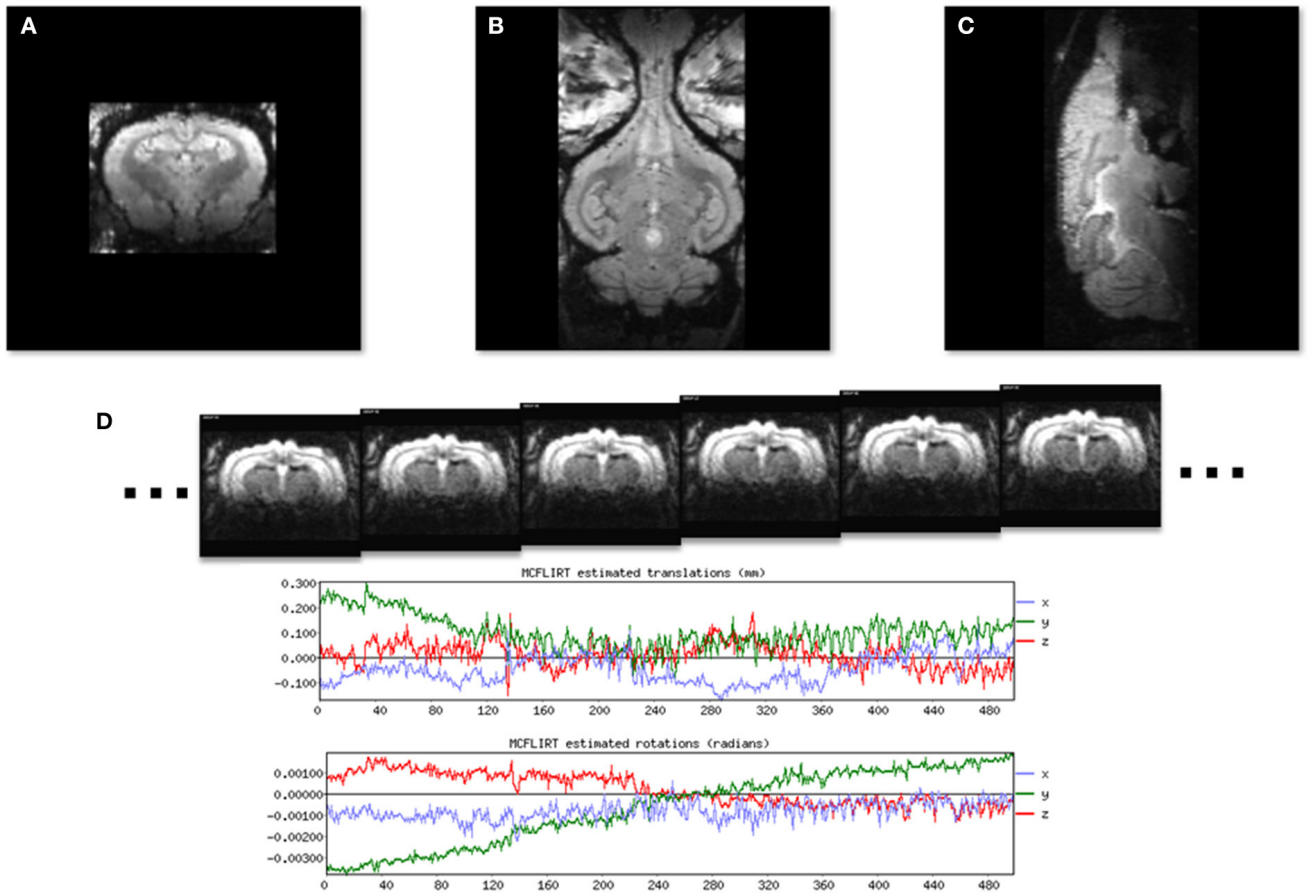
Localizer GRE MR images to show the three standard image planes and the position of the awake subject. **(A)** A coronal slice. **(B)** A horizontal slice. **(C)** A midsagittal slice. **(D)** A time series of six consecutive EPI images used for resting state fMRI from a single image slice. Plots generated by FLIRT (FSL) registration software show rotational and translational displacement of the 500 EPI volumes from the position of the middle volume are <0.3 mm and 0.002 radians.

Images of the brain in each cardinal plane were collected (Figures 3A–C) and were used to adjust the angle of image slices until they were positioned in the stereotaxic planes. Fine tuning of the imaging angles was done by adjusting the imaging parameters. Figure 3D shows 6 consecutive EPI volumes acquired in a 7T MRI scanner (TR = 1,800 ms; TE = 25 ms; Flip Angle = 70; voxel size = 0.65 × 0.65 mm^2^; slice thickness = 1.5 mm; number of slices = 20; matrix = 52 × 68; GRAPPA = 2; echo spacing = 0.25 ms; volumes = 500). Note the two plots below the images which show the stability of the rabbit inside the scanner.

**Figure 4:**
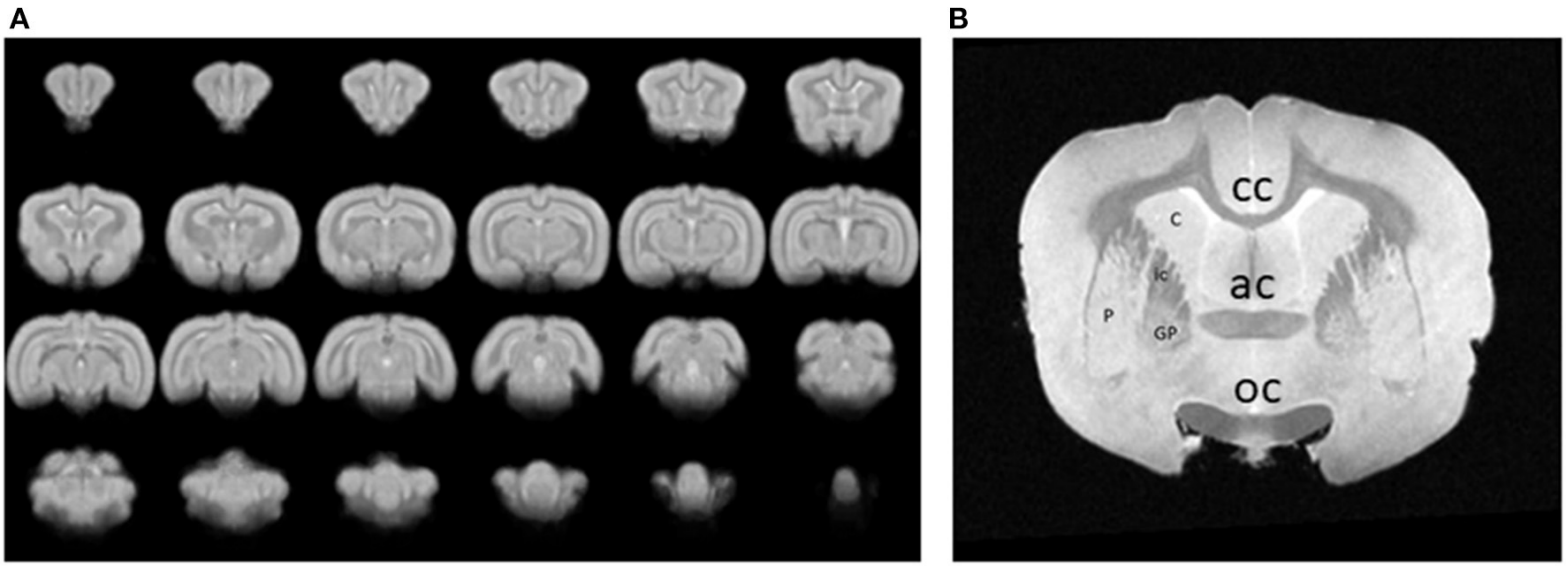
**(A)** A series of 24 coronal images from an awake, restrained rabbit. Rostral is toward the top left, caudal is toward the bottom right. **(B)** An image through the forebrain is shown to highlight the differentiated striatum of the rabbit, i.e., the caudate (C) is separated from the putamen (P) and globus pallidus (GP) by the internal capsule (ic). A differentiated striatum is similar to the structure of the human striatum and unlike the combined caudate-putamen of rodents. The corpus callosum (cc), anterior commissure (ac), and optic chiasm (oc) are also clearly seen in this image slice.

Images were collected for either resting state or event related data after aligning them to the stereotaxic planes. Functional components extracted from the fMRI analysis results were then mapped to a set of anatomical images and then to a rabbit brain template. An example of a series of 24 coronal images from the rabbit brain template, generated by averaging 3D brain images that were acquired from 1 awake, restrained rabbit is shown in Figure 4A. An image through the forebrain is shown (Figure 4B) to highlight the differentiated striatum of the rabbit, i.e., the internal capsule (ic) separates the caudate (C) from the putamen (P) and globus pallidus (GP). The differentiated striatum is similar to the structure of the human striatum and is unlike the combined caudate-putamen of rodents. The corpus callosum (cc), anterior commissure (ac), and optic chiasm (oc) are also clearly seen in this image slice.

## Intrinsically active neuronal networks

**Figure 5:**
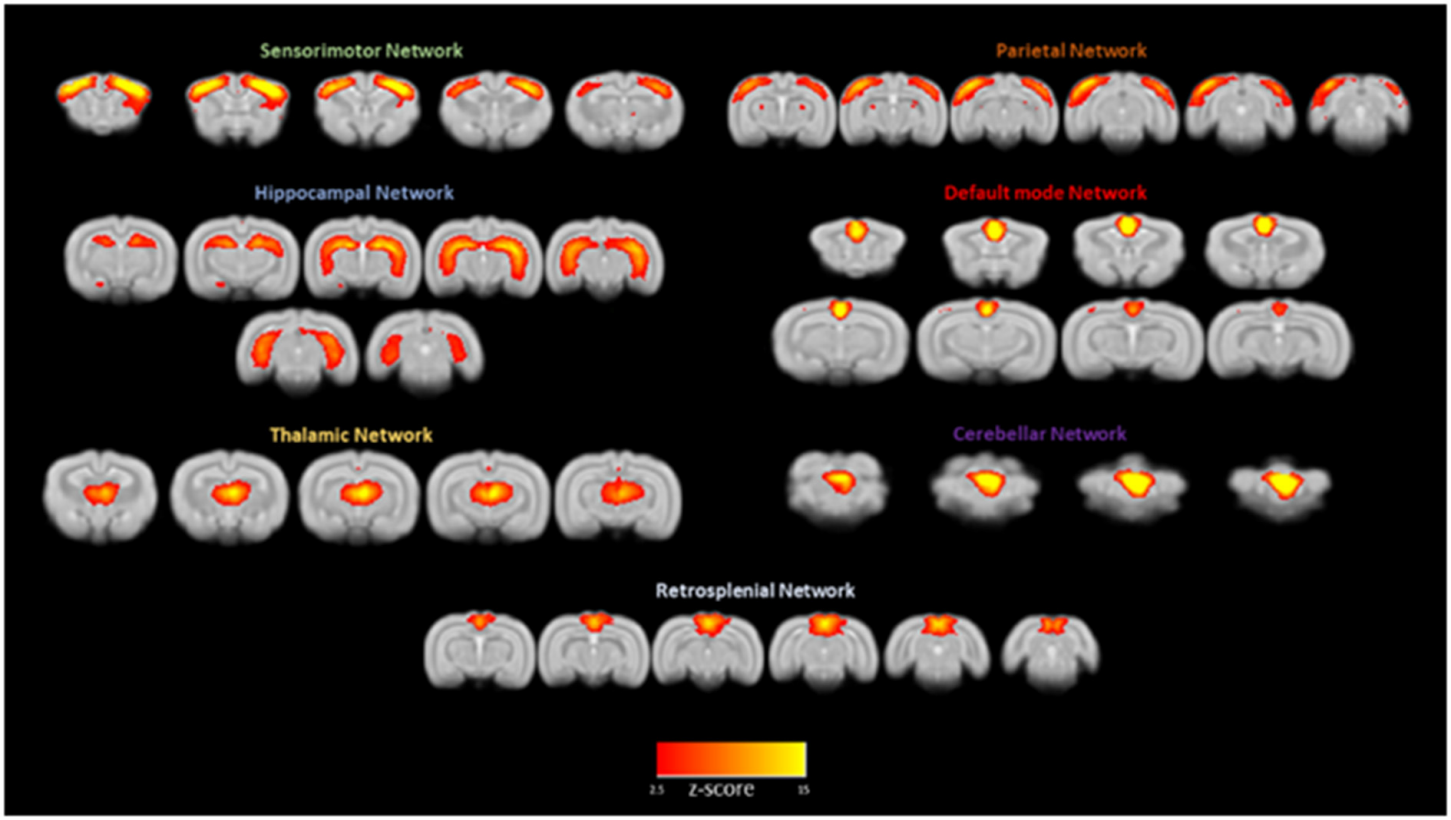
Awake rabbits exhibit functional neuronal networks that are also found in humans. Shown in this figure from Bertolino et al. (2021) are seven networks: sensorimotor, parietal, hippocampal, default mode, thalamic, cerebellar, and retrosplenial. The magnitude of correlated activity for each network is color coded for the Z score of each network. The results confirm what we reported previously (Schroeder et al., 2016).

Intrinsically active functional neuronal networks are especially informative when collected from awake rather than anesthetized animals. Figure 5 shows the main independent functional brain components resulting from a group ICA analysis run on resting state fMRI signals from 12 rabbits. The default mode network is one of the seven networks, and the left and right cerebellar networks were found to be independent of each other. Schroeder et al. (2016) first reported on intrinsic network connectivity in the awake rabbit. Those results are in agreement with our more recent results (Bertolino et al., 2021) with the possible exception of the cerebellar network existing as two independent networks instead of one bilateral cerebellar network as reported by Schroeder et al. (2016).

## Circadian rhythms and light/dark cycles

Another important advantage for working with rabbits is that they are not nocturnal, as are rodents (rabbits are lagomorphs). This is also true for monkeys, but non-human primates are more scarce and more difficult to use for experimental research. While rats and mice tend to have a lower activity level during the normal light phase, rabbits are crepuscular, i.e., they are most active in the hours following sunrise and preceding sunset, and are alert during the rest of the daytime. In our own experience we found that presenting 30 conditioning trials per session tended to lead to better learning than using 50 trials per session in freely moving rats given tone-eye puff pairings (Weiss et al., 1999). The difference was likely related to less opportunity for the rats to sleep during a conditioning session. Rabbits used during the light cycle do not appear to be affected by the pressure and need of sleep. In fact, we typically present 80 trials per session when conditioning rabbits (Rozema et al., 2020).

Having an alert animal during our normal light phase is advantageous for behavioral studies, imaging studies, and animal husbandry routines. Instituting reverse light cycles is an alternative solution, but the change creates challenges at some institutions for husbandry staff and for maintaining the reverse cycle during transport among holding, training, and imaging rooms.

## Eyeblink conditioning as an assay for learning and memory in the magnet

An excellent example of a behavioral paradigm to study simple and more complex associative learning within or outside of the magnet is eyeblink conditioning. The paradigm was developed in the rabbit by Gormezano et al. (1962). They paired a tone with a brief puff of air to the cornea and conditioned the rabbit to extend its nictitating membrane, i.e., a third eyelid, in response to the tone and in anticipation of the airpuff. The minimal essential brain circuitry for this task involves the cerebellum when the airpuff overlaps and co-terminates with the tone conditioned stimulus (Thompson, 2013). Higher order conditioning, particularly “trace” conditioning where there is a stimulus free gap between the end of the tone and onset of the puff requires the hippocampus and forebrain (in addition to the cerebellum) to acquire conditioned responses (Moyer et al., 1990; Kim et al., 1995; Weiss and Disterhoft, 2011). BOLD responses for both trace and delay versions of the task have been recorded from rabbits undergoing conditioning in the magnet (Miller et al., 2003, 2008). Notably, the only movement required of the rabbit is the blink of an eye which has not affected image acquisition, analysis, or interpretation of results in our experiments.

**Figure 6:**
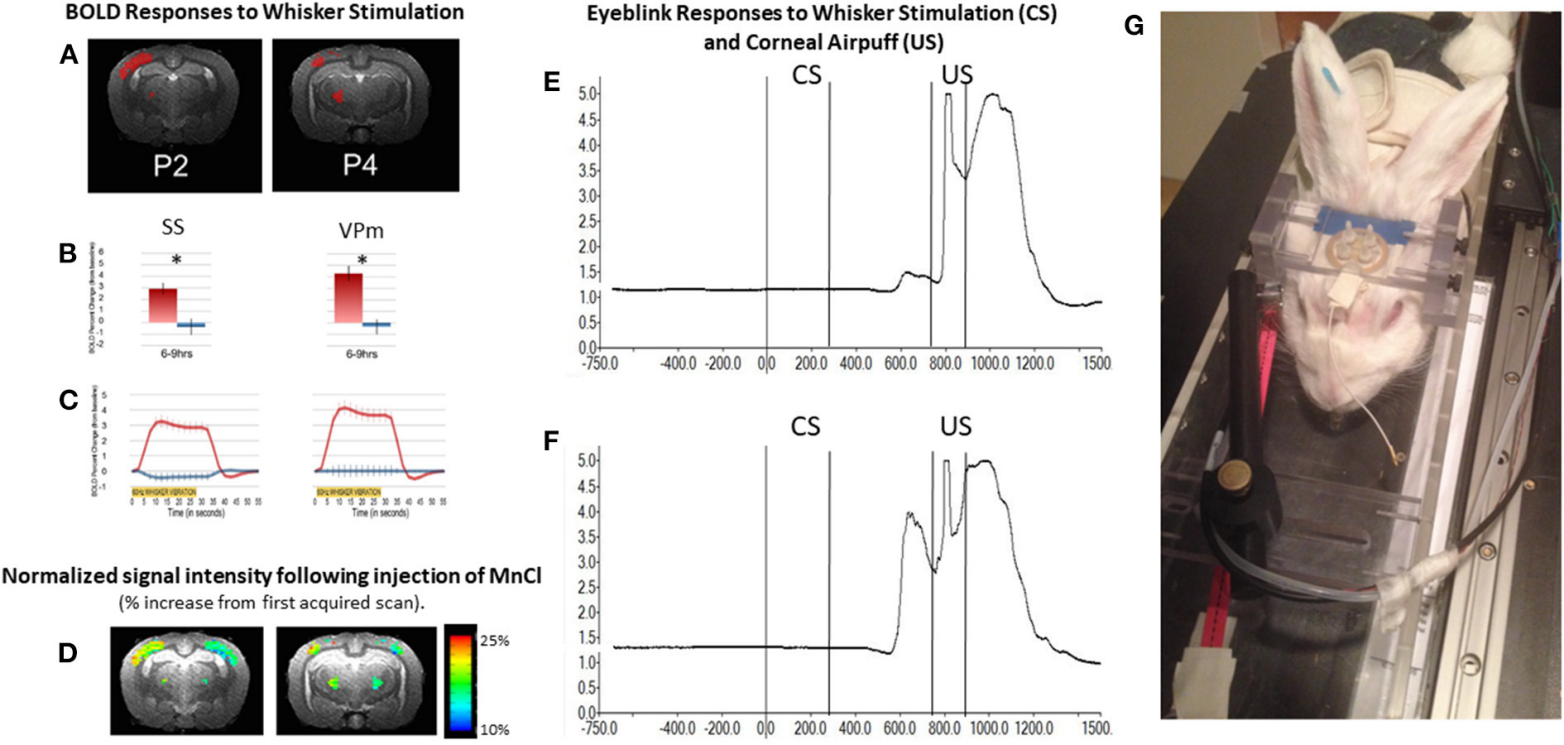
The awake rabbit remains still enough to acquire functional images during sensory stimulation. We first used visual stimulation from green LEDs to activate the contralateral visual cortex and lateral geniculate nucleus (Wyrwicz et al., 2000). More recently we used vibration of the whiskers to evoke activity in the contralateral whisker barrel cortex and the whisker-related ventral posterior medial (VPm) thalamus. **(A)** Activated voxels (in red) from somatosensory cortex near 2 mm posterior to bregma and activated voxels in the ventral posterior medial thalamus (near 4 mm posterior to bregma) in response to contralateral whisker vibration. **(B)** The magnitude of the BOLD response in activated somatosensory (SS) and VPm voxels in **(A)** were quantified. Activation was significantly greater from the contralateral sides (red bars) than from the ipsilateral sides (blue bars). **(C)** The time course of activation to whisker stimulation is shown for both regions from the two hemispheres. **(D)** In addition to analyzing the BOLD response we analyzed the signal intensity of voxels that had accumulated manganese following subcutaneous injection of manganese chloride and whisker stimulation. Manganese enters neurons through voltage gated calcium channels and acts as a surrogate marker for neuronal activation. Accumulation of manganese is detected by MRI due to the fact that Mn2+ shortens the T1-weighted and T2-weighted relaxation times for water molecules in tissue as the molecules realign to the static MR field after a disorienting gradient pulse (Silva et al., 2004; Inoue et al., 2011). Adapted from Schroeder et al. (2016). **(E,F)** respectively, show the change in reflected infrared light during a small and larger conditioned blink response (extension of the nictitating membrane) that were recorded from inside the magnet. Conditioned responses are those that occur in anticipation of the airpuff, US following vibration of the whiskers (CS) on the same side of the face. Fiber optic cables were used to transmit and receive the infrared energy between the eye and an optical amplifier. (**G)** Photograph of a rabbit restrained in the imaging cradle and prepared for vibration of whiskers on right side of face and detection of blinks from the right eye. The cord dangling from the coil to the front of the rabbit would be plugged into the preamplifier.

An example of the BOLD response in whisker barrel cortex of an awake rabbit is shown in Figure 6 at level P2. The BOLD response is also observed in the VPM thalamus at level P4. The results are quantified in panel B and highlight the crossed nature of sensory inputs to the rabbit brain, especially for the whisker system (Gould, 1986), just as the rabbit visual system is about 95% crossed (Giolli and Guthrie, 1969; Hughes and Vaney, 1982). The largely crossed projections to the cortex for the visual and somatosensory systems allows for a direct comparison of stimulated and control conditions.

In addition to analyzing the BOLD response, we analyzed the signal intensity of voxels that had accumulated manganese following subcutaneous injection of manganese chloride and whisker stimulation. Manganese enters neurons through voltage gated calcium channels and acts as a surrogate marker for neuronal activation. Accumulation of manganese is detected by MRI because Mn2+ shortens the T1-weighted and T2-weighted relaxation times for water molecules in tissue as the molecules realign to the static MR field after a disorienting gradient pulse (Silva et al., 2004; Inoue et al., 2011). Although imaging of Mn2+ can be done under anesthesia, we imaged the brain in the awake state so that a direct comparison of the BOLD response and changes in signal intensity due to uptake of Mn2+ could be compared directly.

Last, the rabbit has large ears with easily accessible blood vessels. The ear can be shaved, sterilized, and a needle or catheter can be inserted relatively easily into the marginal ear vein after swaddling a rabbit in a restrainer. Injections of drugs, contrast agents (including magnetic nanospheres), or other approved substances can be made, or blood samples can be collected for monitoring blood chemistry or metabolism.

## Summary and future directions

We have suggested that the rabbit is a very good animal subject to use for fMRI studies of neural activity, largely because of its tolerance for restraint which allows imaging experiments to be done without anesthesia or sedation, and with only minimal habituation to the imaging environment. This avoids interactions with anesthetics and difficulties interpreting results under varying levels of anesthesia (Bajic et al., 2017).

Of course, the rabbit can also be imaged under anesthesia, and doing so would have much less of an impact on structural imaging. In fact, the Ronald laboratory (Chen et al., 2018) has used MRI with fast imaging employing steady state acquisition (FIESTA) with or without susceptibility-weighted post-processing (SWI-FIESTA) and susceptibility weighted imaging with mutli-echo acquisition (SWAN) to image signal voids or hypointensities that reflect amyloid plaques in a rabbit model of Alzheimer's disease based on a diet that includes high cholesterol (0.125 or 0.25% for 24 months). Schreurs et al. (2013) used MRI to examine the effects of 2% cholesterol for 12 weeks and found a significant increase in the size of the third ventricle and a significant decrease in the diameter of major cerebral blood vessels. Jin et al. (2018) also looked at effects of cholesterol in the Japanese White Rabbit (instead of the New Zealand White rabbit) and found that 2% cholesterol plus 0.24 ppm copper (in their drinking water) lowered the ratios of N-acetylaspartate and glutamate to creatine, and increased brain atrophy, similar to what is found in AD.

However, to understand functional aspects of dietary manipulations on learning and memory, and the involvement of neuronal networks in a rabbit model of AD, awake functional imaging is more appropriate. We recently reported the effects of either a high cholesterol diet (2% enrichment) or a combination of 10% saturated fat and 30% fructose on intrinsic functional connectivity and hippocampal dependent memory in the rabbit (Weiss et al., 2022). An independent component analysis (ICA) of 10 networks within the rabbit brain revealed a remarkable loss of functional connectivity among independent network components in rabbits on the cholesterol diet, as compared to rabbits on the control diet. In behavioral tests done outside of the magnet, rabbits on either the cholesterol or the fat/fructose diet were found to recognize familiar objects (a non-hippocampal dependent task) but had impaired memory for the location of an object, a memory system that is dependent on the hippocampus.

Aside from the effects of dietary manipulations on neuronal activity, the rabbit brain can also be manipulated during imaging. Aksenov et al. (2016) injected an adeno-associated viral vector to express channel rhodopsin 2 (pAAV-CaMKIIa:hChR2) and after waiting ~1 month for the virus to be expressed fully, optical stimulation of the injection area evoked a BOLD response and an increase in neuronal activity. Targeting the AAV to inhibitory interneurons should also allow the rabbit brain to be inhibited by optical stimulation *via* an implanted fiber optic cable. Inhibiting neuronal activity during MRI has also been done (in the rat) using ParaMus, a recently developed paramagnetic analog of muscimol (Bricault et al., 2020); this compound should also be effective in the rabbit. Furthermore, brain circuits can be manipulated with molecular specificity during MRI by using magnetic nanospheres coupled to a ligand, as we have done in the rabbit with an antibody (ACU193) to visualize amyloid oligomers (Rozema et al., 2020). Manipulating local brain activity in either an excitatory or inhibitory manner should enable an understanding of the neuronal mechanisms underlying circuit dynamics in the brain.

In summary, we propose that MRI of the awake rabbit is a valuable technique for studying brain activity without complications and interactions due to anesthesia, and without many sessions of habituation, as is required for MRI studies with rats. The natural tolerance of the rabbit to being swaddled for several hours, and its relatively smooth cortex allow good image acquisition and interpretation of results.

## Author contributions

CW prepared rabbits for imaging, co-directed the research, and wrote the first draft of the manuscript. NB and DP collected and analyzed MRI data and edited the manuscript. JD co-directed the research and edited the manuscript. All authors contributed to the article and approved the submitted version.

## Funding

The work in this review article was funded by the National Institutes of Health by grants AG013854, AG050492, and MH047340.

## Conflict of interest

The authors declare that the research was conducted in the absence of any commercial or financial relationships that could be construed as a potential conflict of interest.

## Publisher's note

All claims expressed in this article are solely those of the authors and do not necessarily represent those of their affiliated organizations, or those of the publisher, the editors and the reviewers. Any product that may be evaluated in this article, or claim that may be made by its manufacturer, is not guaranteed or endorsed by the publisher.
